# FDG-PET scan in patients with clinically and/or radiologically suspicious colorectal cancer recurrence but normal CEA

**DOI:** 10.1186/1477-7819-5-64

**Published:** 2007-06-07

**Authors:** Ismet Sarikaya, Mark Bloomston, Stephen P Povoski, Jun Zhang, Nathan C Hall, Michael V Knopp, Edward W Martin

**Affiliations:** 1 Division of Nuclear Medicine, Section of PET, Department of Radiology, The Ohio State University, Columbus, OH, 43210, USA; 2 Division of Surgical Oncology, Department of Surgery, Arthur G. James Cancer Hospital The Ohio State University, Columbus, OH 43210, USA; 3 Richard J. Solove Research Institute and Comprehensive Cancer Center, The Ohio State University, Columbus, OH 43210, USA

## Abstract

**Background:**

Although frequently used for tumor surveillance, the sensitivity of carcinoembryonic antigen (CEA) to detect recurrent colorectal cancer (CRC) is not optimal. Fluorine 18-fluoro-2-deoxy-glucose-positron emission tomography (^18^F FDG-PET) scans promise to improve recurrent CRC detection. We aimed to review PET scans of patients with clinically and/or radiologically suspicious tumor recurrence but normal CEA.

**Methods:**

A retrospective review of an electronic database of 308 patients with CRC who had PET scans was performed. Only PET studies of patients with normal CEAs and suspected tumor recurrence who had pathological verification were selected for further analysis. Thirty-nine patients met the inclusion criteria.

**Results:**

PET was positive in 26 patients (67%) and normal in 13 (33%). Histopathologic evidence of tumor recurrence was seen in 27 of the 39 patients (69%). When correlated with histopathology, PET was true positive in 22 patients, false positive in 4, true negative in 8 and false negative in 5. Overall, the accuracy of PET was 76.9%, negative predictive value (NPV) was 61.5%, and positive predictive value (PPV) was 84.6%. PPV value of PET for liver metastases was 88.8% compared to 73.3% for local recurrence. In two patients with confirmed recurrence, CEA became positive 2 months after PET scan indicating earlier detection of disease with PET. The false positive PET findings were mainly in the bowel and were secondary to acute/chronic inflammation and granulation tissue. In 3 patients with false negative PET, histopathology was consistent with mucinous adenocarcinoma.

**Conclusion:**

PET yields high PPV for recurrent CRC, particularly for liver metastases, in spite of normal CEA levels and should be considered early in the evaluation of patients with suspected tumor recurrence.

## Background

Colorectal cancer (CRC) is the third most common cause of cancer in both men and in women in the United States. The five-year overall survival rates is 64% [1]. Local recurrence rate is relatively high despite radical curative surgery and up to 50% of patients with local recurrence may benefit from surgery.

Early detection and treatment of tumor recurrence is the only hope for long-term survival. Although carcinoembryonic antigen (CEA) is the most frequently used tumor marker, it has low sensitivity in the early detection of recurrent colorectal cancer (CRC). Conventional imaging methods such as Computed Tomography (CT) and Magnetic Resonance Imaging (MR) have limited value in differentiating post-surgical changes from local tumor recurrence. Positron Emission Tomography (PET) and, particularly, Positron Emission Tomography/Computed Tomography (PET/CT) are widely accepted imaging methods in the management of a wide variety of cancers, including CRC. Many studies have demonstrated the value of PET in the detection of CRC recurrence in patients with rising CEA in the post-operative period [2-5]. Studies have also demonstrated the superiority of PET over CT in the detection of local CRC recurrence as well as metastatic disease [6-11]. The role of PET in patients with normal CEA levels, however, is not clear.

In this study, we aimed to analyze the value of FDG-PET scans in patients with normal CEA levels but clinical or radiological findings suspicious for tumor recurrence.

## Methods

A retrospective review of our electronic database of 308 patients with CRC imaged by PET or PET/CT between January 2003 and December 2005 was performed to select and analyze PET scan findings of patients who had normal CEA but clinically and/or radiologically suspicious findings for tumor recurrence. Only patients with correlative histopathological data were included. The institutional review board allowed an exempt retrospective review of the cancer PET database, and informed consent was waived. The normal range for CEA in our clinical laboratory was 0–5.0 ng/mL (chemiluminescent immunoassay). Although the CEA levels were available and normal on all patients at the time of their evaluation for suspicion of recurrent CRC, initial baseline CEA levels taken at a time before their initial treatment for their originally diagnosed CRC were available in only 10 patients (with initial baseline CEA level within normal range in seven patients and elevated in three patients).

PET studies were performed on Siemens Biograph 16 PET/CT and HR plus Siemens CTI PET camera (CTI, Inc, Knoxville, TN). The patients fasted approximately 6 hours prior to intravenous injection of 370–555 MBq (10–15 mCi) of Fluorine 18-fluoro-2-deoxy-glucose (^18^F FDG). Blood glucose levels were checked prior to the injection of ^18^F FDG. Studies were performed only when blood glucose levels did not exceed 150 mg/dL. The imaging started approximately 60 minutes following intravenous injection of ^18^F FDG. For the PET/CT camera, first a scout view was obtained with 30 mAs and 130 kVp followed by a spiral CT scan with 130 mAs, 130 kVp, 5-mm scan width, and 12-mm feed per rotation without any specific breath-holding instructions. No IV or oral contrast was given to the patients for acquisition of the CT images. Imaging area was from skull base to proximal femora. PET scanning was performed immediately after acquisition of the CT images without changing the patient position with 2–4 minutes per bed acquisition time. PET images were corrected for attenuation on the basis of the CT data, and iterative reconstruction algorithms were used for reconstruction. For the PET only camera, image acquisition time of 10 minutes per bed by using 40% transmission and 60% emission protocol was used. PET images were corrected for attenuation on the basis of the transmission image data, and iterative reconstruction algorithms were used for reconstruction. PET images were evaluated by two board-certified nuclear medicine physicians (IS and NCH). PET scan was considered positive or suspicious when abnormal non-physiologic metabolic activity was identified. Focal hypermetabolic activity within the liver greater than adjacent normal liver was considered abnormal. Isometabolic liver lesions (metabolic activity equal to liver) were only identified with the help of CT in patients with PET/CT scan. Diffuse mild activity in the bowel was considered normal physiologic uptake. Quantification of the tumor metabolic activity was obtained using the Standardized Uptake Value (SUV) normalized to body weight. Mean ± SD of maximum-pixel SUV (SUV_max_) of the lesions were calculated. The significance of SUV_max _between false positive and true positive lesions were compared by t-test. A P-value of less than or equal to 0.05 was considered statistically significant.

## Results

A total of 369 PET studies (PET: 183, from January 2003 to March 2005 and combined PET/CT: 186, from April 2005 to December 2006) in 308 patients with CRC were reviewed. Only patients with clinical and/or radiological suspicion of tumor recurrence but normal CEA who have histopathological evaluation following PET scan were selected for further analysis. Thirty-nine patients met the inclusion criteria. The PET studies were performed by PET scan alone in 27 patients and by combined PET/CT scan in 12 patients. PET was ordered in these patients because of suspicious or equivocal lesions on CT in 17, on barium study in two, or on history and physical exam in 20. The characteristics of the patients are summarized in Table 1. At the time of suspected recurrent CRC, the mean age was 55 with a near-equal gender distribution. The majority of patients had undergone surgical resection alone with the remainder having chemotherapy and/or radiotherapy prior to or following resection. In two patients receiving chemoradiation, resection of the primary was not undertaken. Surgical exploration was undertaken within one month of PET scan in 37 patients and within two months of PET scan in the other two.

**Table 1:** Patient Characteristics

**Characteristics**	**Patients**
Mean age [range (year)]	55 (33–91)
Gender	
Males	18
Females	21
Mean time interval from initial treatment to PET scan [range (months)]	24 (3–92)
Mean time interval from last treatment to PET scan [range (months)]	25 (3–92)

**Figure 1:**
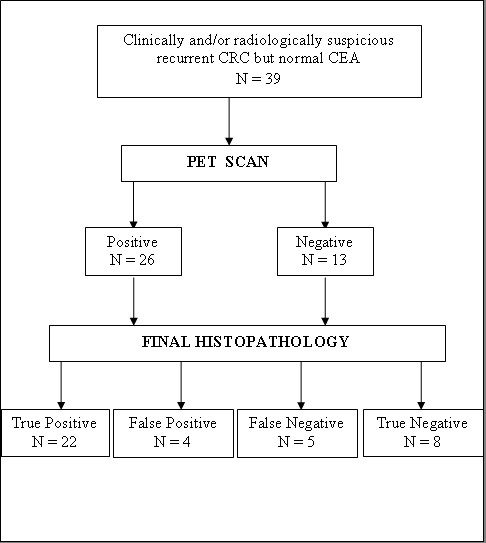
PET scan and final pathology results of the patients who have clinically and/or radiologically suspicious tumor recurrence but normal CEA.

PET scan was considered normal in 13 (33%) and positive in 26 (67%). At exploration, 27 (69%) patients were found to have histopathologic evidence of tumor recurrence. Of 26 positive PET scans, 22 were true positive and four were false positive. Eight patients were found to have true negative PET scans while five were false negative (Figure 1). In three patients with a false negative PET scan, tumor was mucinous adenocarcinoma and in the other two it was moderately differentiated adenocarcinoma. The overall accuracy of PET was 76.9% with a sensitivity of 81.4%, specificity of 66.6%, positive predictive value (PPV) of 84.6%, and negative predictive value (NPV) of 61.5%.

**Figure 2:**
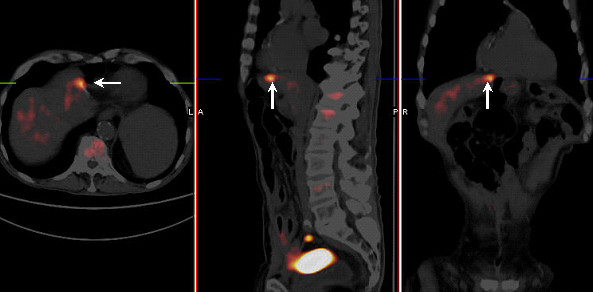
Transaxial, sagittal, and coronal PET/CT fusion images in a patient with prior left lobectomy. PET scan demonstrated a focal hypermetabolic activity within the liver at resectin margin with SUV_maxof _3.1 (arrow). Pathology demonstrated focal foreign body reaction with necrosis, organizing inflammation and fibrosis secondary to prior surgery.

**Figure 3:**
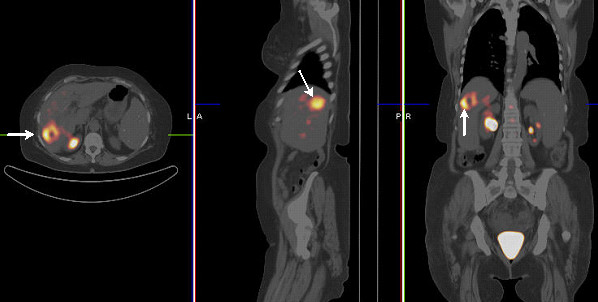
Transaxial, sagittal, and coronal PET/CT fusion images demonstrating a ring-shaped liver lesion (arrow) with increased metabolic activity (SUV_max_:5.5) surrounding a photopenic center in a patient previously treated with Yittrium-90 microspheres. CEA became positive two months after PET and subsequent pathology demonstrated tumor recurrence as well as nodal metastasis.

In the 26 patients with positive PET scans, PET detected a total of 36 lesions, 32 of which were resected. Histopathological findings in these patients are shown in Table 2. PPV value of PET was highest for liver metastases (88.8%) compared to 73.3% for luminal (i.e. anastomotic) recurrences. All recurrences and metastases were consistent with adenocarcinomas with one demonstrating mucinous features. The exact lesion size was discernable in six patients and ranged from 10 to 30 mm. In two patients with pathology proven adenoma with dysplasia in rectum, PET was accepted as true positive for these lesions due to their pre-malignant nature. Pathology for one patient (patient number 24 in Table 2) with a false positive PET scan in the liver (SUV_max _of 3.1) showed focal foreign body reaction with necrosis, organizing inflammation, and fibrosis related to previous surgery (Figure 2). The remaining false positive cases were mainly in the bowel and were secondary to acute/chronic inflammation and granulation tissue. In patients with a false positive PET scan, the time interval between last treatment and PET was 42 months (range:11–96 months). In two cases with a positive PET scan (patient number 8 and 15 in Table 2), CEA became elevated two months after PET scan and subsequent pathology demonstrated tumor recurrence. In one of these two patients, PET showed increased activity in the liver with central photopenia (Figure 3) following treatment with Yttrium-90 microspheres. In one patient (patient number 26 in Table 2) with a false positive PET scan in the rectum, PET scan also demonstrated a bone lesion. Follow-up evaluation demonstrated progression in the bone lesion consistent with metastasis. In two patients with pathology-proven intra-abdominal tumor recurrence, PET also demonstrated mediastinal uptake (patient number 9 and 19 in Table 2). These lesions remained stable on follow-up PET and CT images.

**Table 2:** PET and histopathological findings in 26 patients with positive PET scan

**No**	**PET/Location**	**SUV**	**Pathology**	**Location of RCRC**
1*	Rectum	5.8	Tubulovillous adenoma	Rectum
2*	Rectum	8	MD invasive adeno ca	Rectum/P LN
3*	Liver	3.4	Colon SD adeno ca met	Liver
4*	Lung	8.6	Colon adeno ca met	Lung
5*	Rectum	1.8	MD mucinous adeno ca	Rectum
6*	Liver	8.2	Colon adeno ca met	Liver
7	Liver	6.3	MPD invasive adeno ca	Liver
8	Liver	5.5	MD invasive adeno ca	Liver
9	Liver	6.9	MPD invasive adeno ca	Liver/sigmoid/A LN
	Mediastinum	3.8	No biopsy	
10	Lung	1.8	Colon IT adeno ca met	Lung
	Rectum	4.5	Inflammation/granulation	
11	Rectum	4.8	MD invasive adeno ca	Rectum
	AP LN	6.3	No biopsy	
12	Multiple liver	6	Colon adeno ca met	Liver
13	Liver	3.8	Colon adeno ca met	Liver/AP LN/Ovary
	AP LN	3.6	Colon adeno ca met	
14	Liver	3.1	MD adeno ca met	Liver
15	Cecum	1.8	MD adeno ca	Cecum/A LN
16	Portal	2.9	Colon adeno ca met	Portal/Stomach wall
	Upper A	6.1	Colon adeno ca met	
17	Sigmoid/Rectum	8.2	Adeno ca	Sigmoid/Rectum
18	Rectum	5.4	MD adeno ca	Rectum
19	Right colon	11.4	MD adeno ca	Right Colon/A LN/Liver
	A LN	6.3	Colon adeno ca met	
	Mediastinium	6.3	No biopsy	
20	Rectum	12	MD adeno ca	Rectum
21	Rectum	3.2	Tubular adenoma	Rectum
22	Rectum	4.3	MD mucinous adeno ca	Rectum/Duodenum
	Upper A	4	MD mucinous adeno ca	
23*	Splenic Flexure	6.5	Acute inflammation	
24*	Liver	3.1	Focal foreign body reaction	
	A LN	3.3	Acute inflammation	
25*	Rectum	16.7	Acute inflammation	
26*	Rectum	6.4	Acute inflammation	
	Bone	4.1	No biopsy	

RCRC: Recurrent CRC, MD: Moderately differentiated, MPD: Moderate to poorly differentiated, SD: Squamoid differentiation, IT: Intestinal type, LN: Lymph nodes, A: Abdominal, P: Pelvic, AP: Abdominopelvic, *: Studies done on PET/CT camera.

In all patients with negative PET and negative pathology results, follow-up CEA values were available. In six of these patients, follow-up CEA values were normal (mean follow-up time 18.8 months, range: 4–41 months). In two patients, follow-up CEA level increased (mean follow-up time 3 months, range: 2–6 months).

PET and PET/CT results were evaluated separately. PET was true positive in 16 patients, false negative in three patients, and true negative in eight patients. PET/CT was true positive in six patients, false positive in four patients, and false negative in two patients. There was no false positive result with PET and no true negative result with PET/CT. The accuracy of PET and PET/CT was 88.8% and 50%, respectively.

Mean ± SD of SUV_max _of true positive and false positive lesions are shown in Table 3. The mean SUV_max _in all true positive lesions tended to be lower than in all false positive lesions, though this did not reach statistical significance (5.51 ± 2.73 vs. 6.75 ± 5.1, p = 0.58). False positive PET findings associated with bowel showed a trend towards higher SUV_max _compared to true positive lesions, but this was not statistically significant (8.5 ± 5.5 vs. 6.06 ± 3.5, p = 0.45).

**Table 3:** Mean SUV_max _± standard deviation (SD) in true positive and false positive PET lesions based upon location of PET positive lesions.

	**True Positive PET**	**SUV_max _(range)**	**False Positive PET**	**SUV_max _(range)**
Liver	8	5.4 ± 1.8 (3.1–8.2)	1	3.1
Bowel	11	6.06 ± 3.5 (1.8–12)	4	8.5 ± 5.5 (4.5–16.7)
Lymph nodes	3	4.2 ± 1.7 (2–9–6.3)	1	3.3
Lungs	2	5.25 ± 4.87 (1.8–8.7)	0	N/A
Other intra-abdominal	2	5.05 ± 1.48 (4–6.1)	0	N/A
**Total**	**26**	**5.51 ± 2.73 (1.8–12)**	**6**	**6.75 ± 5.1 (3.1–16.7)**

N/A – not applicable

## Discussion

Approximately 70% of patients with newly diagnosed CRC are suitable for a curative resection, but up to 50% of patients recurrence develops usually within two years of surgery [12-14]. Ten to 50% of local recurrences may be suitable for surgical resection [12-20]. In patients with liver metastasis hepatic resection in properly selected patients offers up to a 30% chance of cure [21]. Early detection and prompt treatment of recurrence improves survival. Although CEA is used frequently in the post-operative follow-up period, its sensitivity for early detection of CRC recurrence is less than desirable [22]. Moertel et al. reported that CEA has a sensitivity of 59% in the detection of CRC recurrence [23]. CEA is also not specific for colorectal cancer. A wide variety of non-neoplastic conditions, such as smoking and liver and gastrointestinal diseases, may cause elevation in CEA. The use of PET as part of the diagnostic work-up of patients with rising CEA is well defined. However, there is a paucity of data concerning its role in patients with suspected CRC recurrence and normal CEA. We have shown that PET can be reliably applied in such patients to allow for earlier detection and management of recurrent CRC.

PET is a functional/metabolic imaging technique which has been widely used in the diagnosis, staging, and management of a wide variety of tumors. The most commonly used PET agent in oncology is ^18^F FDG, a positron-labeled non-physiologic analog of glucose. Malignant tumors avidly accumulate FDG because of accelerated glucose metabolism and increased rate of glucose transport and utilization in malignant cells. FDG in the blood is transported into the cells via glucose transporters and phosphorylated to FDG-6-phosphate by hexokinase. This is thought to occur more readily in tumors due to overexpression of the glucose transporters GLUT1 and GLUT3 and higher levels of hexokinase in malignant cells [24]. Because Glucose-6-phosphatase enzyme is low in most of the tissues and tumors, FDG-6-phosphate cannot be dephosphorylated to FDG. Therefore, FDG-6-phosphate cannot cross the cell membrane and is trapped in the cell. As well, FDG-6-phosphate cannot be utilized in the metabolic steps of glycolysis resulting in accumulation of the radioactive tracer.

Many studies have demonstrated that FDG-PET has high sensitivity and specificity in the detection of tumor recurrence in patients with rising tumor markers in the absence of a known source by anatomical imaging methods [2-5]. A meta-analysis determined the overall sensitivity of 97% and specificity of 76% for FDG PET for detecting recurrent CRC [25]. Studies have also demonstrated that FDG PET is more sensitive than CT for the detection of recurrent CRC [6-11]. PET was found to be superior to CT in the differentiation of fibrotic scar tissues from locally recurrent tumor [26]. The accuracy of PET for locally recurrent disease was reported as 95% which was superior to pelvic-CT (65%) [27]. PET is more sensitive than CT in detecting liver metastases and defining the number of the lobes involved. Arulapalam et al. reported the sensitivity of PET and CT as 100% and 45%, respectively, in the detection of liver metastasis [6]. Similar to CT, MRI also has limitations in the differentiation of fibrotic scar from local recurrence. Although there is increasing use of dynamic contrast-enhanced MR, it is not clear yet whether it is superior to PET in the detection of recurrence. Contrast-enhanced liver MRI and whole-body FDG-PET were comparable in the detection of patients with liver metastases [28]. PET provided additional information about extrahepatic disease. PET is a valuable imaging method to differentiate isolated resectable recurrence from disseminated metastatic disease to select patients who would benefit from surgery [29]. Studies have also compared PET and PET/CT in the detection of CRC recurrence. The sensitivity, specificity and overall accuracy of PET were 80%, 69%, and 75%, respectively, compared with 89%, 92%, and 90%, respectively, for PET/CT [30]. Another study demonstrated superiority of PET/CT over PET in differentiating malignant from benign lesions and physiologic activities [31]. The most common cause for false-positive interpretation of PET findings was physiologic FDG uptake in pelvic organs.

In our study, histopathological analysis following PET scan demonstrated tumor recurrence or metastasis in 27 of 39 patients who have normal CEA but clinically and/or radiologically suspicious tumor recurrence. Twelve patients were tumor free. PET was true positive in 22 patients and true negative in eight patients. The accuracy of PET was 76.9%. Our results demonstrated that PET was most accurate for liver metastases with a positive predictive value 88.8%. For local recurrence, PET had PPV of 73.3%. PET was false negative in five patients. In three of these patients pathology was consistent with mucinous carcinoma with signet cells. Consistent with our findings, it has been reported that the sensitivity of FDG-PET imaging for detection of mucinous carcinoma is significantly lower than in nonmucinous carcinoma (58% and 92%, respectively) [8]. PET was false positive in four patients. False positive PET lesions were mainly in the bowel and were secondary to infectious or inflammatory/granulomatous processes. False-positive findings with FDG-PET in colorectal region are not uncommon [32]. Increased FDG uptake is observed at recent incision and biopsy sites, around drainage tubes and catheters, and at colostomy sites, as well as in association with colitis, abscesses, fistulas, diverticulitis, and in some benign adenomas. This is because of increased glucose use in activated macrophages, neutrophils, and fibroblasts within infectious, inflammatory, and granulomatous tissues. Normal FDG uptake in the gastrointestinal tract may also cause difficulty to differentiate normal physiologic uptake from recurrent tumor.

There are many factors affecting the serum concentration of CEA, including tumor location, proximity to large vessels, degree of de-differentiation, access to portal circulation, tumor distribution, and tumor burden. Previously Moertel et al reported the inadequacy of CEA for early detection of tumor recurrence [23]. Only 25% of the patients had abnormal CEA levels although most patients had symptoms of recurrence for several months in their study. In two of our patients, CEA became positive 2 months after PET scan, suggesting that PET can show tumor recurrence earlier than CEA elevation. In our study, the lesion size was measured in focal solitary liver metastases in six patients. The tumor size ranged from 10 mm to 30 mm. However, our data is not sufficient to find the smallest tumor volume where PET can be positive while CEA negative. There are also many other factors in addition to tumor size affecting CEA level. However, we assume that given directly imaging the tumor and high glucose metabolism in tumor tissue, as well as current high-resolution PET/CT cameras, it is expected that FDG-PET may detect tumor recurrence before significant increase in CEA level.

Although PET facilitates the evaluation of metabolic characteristics of tumors, it is limited in its ability to visualize anatomical structures. A PET/CT camera is the combination of PET and CT cameras which allows more accurate registration of metabolic findings in tumor with anatomical findings, adding further information to the diagnosis and staging of tumors. In PET/CT cameras, CT is also used for attenuation correction of PET images which significantly reduces imaging time. In our study, the accuracy of PET/CT was found to be lower than PET alone. However, given the smaller number of exams performed by PET/CT than by PET alone, this determination of accuracy may not reliably reflect the actual accuracy of PET/CT versus PET alone.

Our study had several potential limitations. The first potential limitation was our small sample size which may have limited the robustness of our study in terms of statistics. The second potential limitation was the retrospective nature of our study. Because of its retrospective nature, we were unable to obtain baseline clinical and laboratory data in some of the patients. The third potential limitation was the pooled nature of the PET and PET/CT data, given that different attenuation correction algorithms were used in these two imaging methods. However, this issue would be of more concern had we compared PET SUVmax to PET/CT SUVmax in the same given patient. In defense of this third potential limitation, there is recently published study pooling PET and PET/CT SUV results [33]. The fourth potential limitation was our inability to use oral contrast for the combined PET/CT studies, since some data is available which demonstrates that low density oral contrast administration during combined PET/CT studies further increases accuracy.

## Conclusion

Although PET is commonly used to localize tumor recurrence in cases with elevated CEA, our results indicate that PET is also valuable to detect tumor recurrence in selected cases who have normal CEA but clinically and/or radiologically suspicious tumor recurrence. The PPV of PET is high, particularly in detecting liver metastases. While PET may not be practical for routine surveillance for all patients with CRC, it should be utilized in select cases where CEA is not reliable, such as tumors known to not secrete CEA. PET should also be utilized at the first sign of suspected recurrence to determine the extent of disease.

## Competing interests

The author(s) declare that they have no competing interests.

## Authors' contributions

**IS **designed the current study and collected the data. She organized, wrote, and revised the manuscript. **MB **was also involved in study design and the data collection. He performed the analyses of all the data. He assisted in the organization, writing, and editing of all aspects of this manuscript. **SPP**, **NCH**, and **MVK **assisted in the writing and editing of all aspects of this manuscript. **EWM **was the supervising senior physician for the entire project and assisted in the writing and editing of all aspects of this manuscript. All the authors have approved the final version of this manuscript.

## References

[B1] Jemal A, Siegel R, Ward E, Murray T, Xu J, Thun MJ (2007). Cancer statistics, 2007. CA Cancer J Clin.

[B2] Zervos EE, Badgwell BD, Burak WE, Arnold MW, Martin EW (2001). Fluorodeoxyglucose positron emission tomography as an adjunct to carcinoembryonic antigen in the management of patients with presumed recurrent colorectal cancer and nondiagnostic radiologic workup. Surgery.

[B3] Flamen P, Hoekstra OS, Homans F, Van Cutsem E, Maes A, Stroobants S, Peeters M, Penninckx F, Filez L, Bleichrodt RP, Mortelmans L (2001). Unexplained rising carcinoembryonic antigen (CEA) in the postoperative surveillance of colorectal cancer: the utility of positron emission tomography (PET). Eur J Cancer.

[B4] Maldonado A, Sancho F, Cerdan J, Lozano A, Mohedano N, Jimenez J, Moya F, Zomeno M (2000). 16. FDG-PET in the Detection of Recurrence in Colorectal Cancer Based on Rising CEA Level. Experience in 72 Patients. Clin Positron Imaging.

[B5] Flanagan FL, Dehdashti F, Ogunbiyi OA, Kodner IJ, Siegel BA (1998). Utility of FDG-PET for investigating unexplained plasma CEA elevation in patients with colorectal cancer. Ann Surg.

[B6] Arulampalam T, Costa D, Visvikis D, Boulos P, Taylor I, Ell P (2001). The impact of FDG-PET on the management algorithm for recurrent colorectal cancer. Eur J Nucl Med.

[B7] Hung GU, Shiau YC, Tsai SC, Chao TH, Ho YJ, Kao CH (2001). Value of 18F-fluoro-2-deoxyglucose positron emission tomography in the evaluation of recurrent colorectal cancer. Anticancer Res.

[B8] Whiteford MH, Whiteford HM, Yee LF, Ogunbiyi OA, Dehdashti F, Siegel BA, Birnbaum EH, Fleshman JW, Kodner IJ, Read TE (2000). Usefulness of FDG-PET scan in the assessment of suspected metastatic or recurrent adenocarcinoma of the colon and rectum. Dis Colon Rectum.

[B9] Valk PE, Abella-Columna E, Haseman MK, Pounds TR, Tesar RD, Myers RW, Greiss HB, Hofer GA (1999). Whole-body PET imaging with [18F]fluorodeoxyglucose in management of recurrent colorectal cancer. Arch Surg.

[B10] Ogunbiyi OA, Flanagan FL, Dehdashti F, Siegel BA, Trask DD, Birnbaum EH, Fleshman JW, Read TE, Philpott GW, Kodner IJ (1997). Detection of recurrent and metastatic colorectal cancer: comparison of positron emission tomography and computed tomography. Ann Surg Oncol.

[B11] Delbeke D, Vitola JV, Sandler MP, Arildsen RC, Powers TA, Wright JK, Chapman WC, Pinson CW (1997). Staging recurrent metastatic colorectal carcinoma with PET. J Nucl Med.

[B12] Galandiuk S, Wieand HS, Moertel CG, Cha SS, Fitzgibbons RJ, Pemberton JH, Wolff BG (1992). Patterns of recurrence after curative resection of carcinoma of the colon and rectum. Surg Gynecol Obstet.

[B13] Cass AW, Million RR, Pfaff WW (1976). Patterns of recurrence following surgery alone for adenocarcinoma of the colon and rectum. Cancer.

[B14] Olson RM, Perencevich NP, Malcolm AW, Chaffey JT, Wilson RE (1980). Patterns of recurrence following curative resection of adenocarcinoma of the colon and rectum. Cancer.

[B15] Turk PS, Wanebo HJ (1993). Results of surgical treatment of nonhepatic recurrence of colorectal carcinoma. Cancer.

[B16] Pilipshen SJ, Heilweil M, Quan SH, Sternberg SS, Enker WE (1984). Patterns of pelvic recurrence following definitive resections of rectal cancer. Cancer.

[B17] Welch JP, Donaldson GA (1978). Detection and treatment of recurrent cancer of the colon and rectum. Am J Surg.

[B18] Gunderson LL, Sosin H (1974). Areas of failure found at reoperation (second or symptomatic look) following "curative surgery" for adenocarcinoma of the rectum. Clinicopathologic correlation and implications for adjuvant therapy. Cancer.

[B19] Rao AR, Kagan AR, Chan PM, Gilbert HA, Nussbaum H, Hintz BL (1981). Patterns of recurrence following curative resection alone for adenocarcinoma of the rectum and sigmoid colon. Cancer.

[B20] Rich T, Gunderson LL, Lew R, Galdibini JJ, Cohen AM, Donaldson G (1983). Patterns of recurrence of rectal cancer after potentially curative surgery. Cancer.

[B21] Kelly CJ, Daly JM (1992). Colorectal cancer. Principles of postoperative follow-up. Cancer.

[B22] Moertel CG, Schutt AJ, Go VL (1978). Carcinoembryonic antigen test for recurrent colorectal carcinoma. Inadequacy for early detection. JAMA.

[B23] Moertel CG, Fleming TR, Macdonald JS, Haller DG, Laurie JA, Tangen C (1993). An evaluation of the carcinoembryonic antigen (CEA) test for monitoring patients with resected colon cancer. JAMA.

[B24] Burt BM, Humm JL, Kooby DA, Squire OD, Mastorides S, Larson SM, Fong Y (2001). Using positron emission tomography with [(18)F]FDG to predict tumor behavior in experimental colorectal cancer. Neoplasia.

[B25] Huebner RH, Park KC, Shepherd JE, Schwimmer J, Czernin J, Phelps ME, Gambhir SS (2000). A meta-analysis of the literature for whole-body FDG PET detection of recurrent colorectal cancer. J Nucl Med.

[B26] Takeuchi O, Saito N, Koda K, Sarashina H, Nakajima N (1999). Clinical assessment of positron emission tomography for the diagnosis of local recurrence in colorectal cancer. Br J Surg.

[B27] Schiepers C, Penninckx F, De Vadder N, Merckx E, Mortelmans L, Bormans G, Marchal G, Filez L, Aerts R (1995). Contribution of PET in the diagnosis of recurrent colorectal cancer: comparison with conventional imaging. Eur J Surg Oncol.

[B28] Sahani DV, Kalva SP, Fischman AJ, Kadavigere R, Blake M, Hahn PF, Saini S (2005). Detection of liver metastases from adenocarcinoma of the colon and pancreas: comparison of mangafodipir trisodium-enhanced liver MRI and whole-body FDG PET. Am J Roentgenol.

[B29] Staib L, Schirrmeister H, Reske SN, Beger HG (2000). Is (18)F-fluorodeoxyglucose positron emission tomography in recurrent colorectal cancer a contribution to surgical decision making?. Am J Surg.

[B30] Votrubova J, Belohlavek O, Jaruskova M, Oliverius M, Lohynska R, Trskova K, Sedlackova E, Lipska L, Stahalova V (2006). The role of FDG-PET/CT in the detection of recurrent colorectal cancer. Eur J Nucl Med Mol Imaging.

[B31] Even-Sapir E, Parag Y, Lerman H, Gutman M, Levine C, Rabau M, Figer A, Metser U (2004). Detection of recurrence in patients with rectal cancer: PET/CT after abdominoperineal or anterior resection. Radiology.

[B32] Gutman F, Alberini JL, Wartski M, Vilain D, Le Stanc E, Sarandi F, Corone C, Tainturier C, Pecking AP (2005). Incidental colonic focal lesions detected by FDG PET/CT. AJR.

[B33] Schoder H, Herrmann K, Gonen M, Hricak H, Eberhard S, Scardino P, Scher HI, Larson SM (2005). 2-[18F]fluoro-2-deoxyglucose positron emission tomography for the detection of disease in patients with prostate-specific antigen relapse after radical prostatectomy. Clin Cancer Res.

